# Giant anal condyloma (giant condyloma acuminatum of anus) after allogeneic bone marrow transplantation associated with human papillomavirus: a case report

**DOI:** 10.1186/1752-1947-9-9

**Published:** 2015-01-19

**Authors:** Jin-Soo Hyun, Gee-Bum Kim, Byung-Seok Choi, Min-Sung Kim, Sang-Gon Park

**Affiliations:** 1 Department of Internal Medicine, Chosun University Hospital, 365 Pilmun-daero, Dong-gu, Gwangju, 501-717 Republic of Korea; 2 Department of Medicine, Graduate School of Chosun University, Misigain Skin Clinic, 6th neopraza building 936 sau-dong, Gimpo, Gyeonggi Republic of Korea; 3 Department of Medicine, Graduate School of Chosun University, 309 Pilmun-daero, Dong-gu, Gwangju 501-717 Republic of Korea; 4 Department of Dermatology, Chosun University Hospital, 365 Pilmun-daero, Dong-gu, Gwangju 501-717 Republic of Korea; 5 Department of Internal Medicine, Hemato-oncology, 365 Pilmun-daero, Dong-gu, Gwangju 501-717 Republic of Korea

**Keywords:** Condyloma, HPV, Allogenic bone marrow transplantation, Anus condyloma

## Abstract

**Introduction:**

Condyloma acuminatum are caused by human papillomavirus. Giant condyloma acuminatum is a locally invasive, destructive, and large sized mass. Risk factors for the development of giant condyloma acuminatum include an immunodeficient state, such as human immunodeficiency virus infection, post-organ transplantation, or post-allogeneic bone marrow transplantation. However, reports of giant condyloma after bone marrow transplantation are extremely rare (0.3 to 1.3%). The standard treatment for giant condyloma acuminatum is recommended as wide surgical resection due to its high rate of success and low rate of recurrence.

**Case presentation:**

A 31-year-old Korean man presented to our hospital with anal discomfort for more than one month due to a protruding mass. He had a history of BCR-ABL-positive acute lymphoblastic leukemia and had undergone an allogenic stem cell transplantation. Gross findings revealed a large perianal cauliflower-like mass over 7cm in size with invasion of the anal orifice. He was diagnosed with giant anal condyloma occurring after an allogeneic bone marrow transplantation. However, we achieved successful treatment using a combination of topical podophyllin and cryotherapy and transanal surgical excision, followed by bleomycin irrigation.

**Conclusions:**

We report an extremely rare case of giant condyloma acuminatum of anus due to human papillomavirus type six in a patient with acute lymphoblastic leukemia following an allogeneic bone marrow transplantation. The tumor was successfully treated with a combination of topical podophyllin and cryotherapy and transanal surgical excision, followed by bleomycin irrigation.

## Introduction

Rapid proliferation of human papillomavirus (HPV) in immunodeficiency patients leads to larger size and locally invasive tumors with or without dysplasia. Risk factors for giant condyloma acuminatum (GCA) include an immunodeficient state, such as human immunodeficiency virus (HIV) infection, post-organ transplantation, and post-allogeneic bone marrow transplantation. However, reports of giant condyloma after bone marrow transplantation are extremely rare (0.3 to 1.3%) [1–3]. There are various treatment methods including wide surgical resection, however, no definitive therapeutic guideline has yet been established [4, 5].

## Case presentation

**Figure 1:**
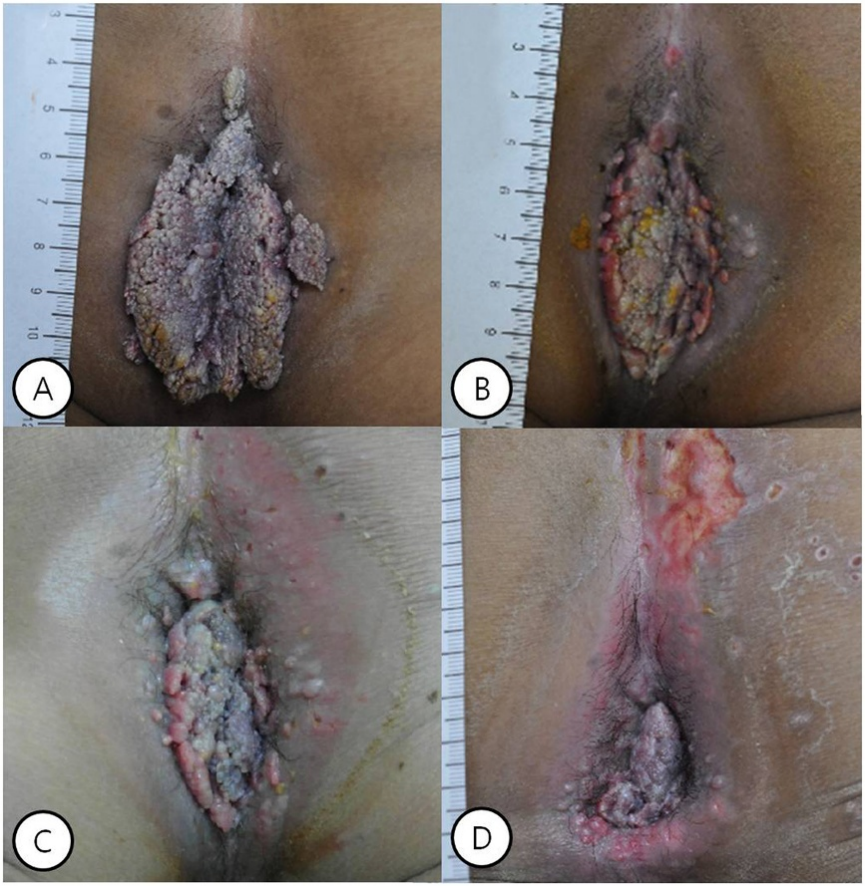
**Gross finding of giant condyloma acuminatum of anus. (A)** there was large cauliflower-like mass over 7cm in size with invasion of the anal orifice in perianal lesion. **(B)** One week after local therapy, **(C)** two weeks after local therapy, **(D)** four weeks after local therapy.

**Figure 2:**
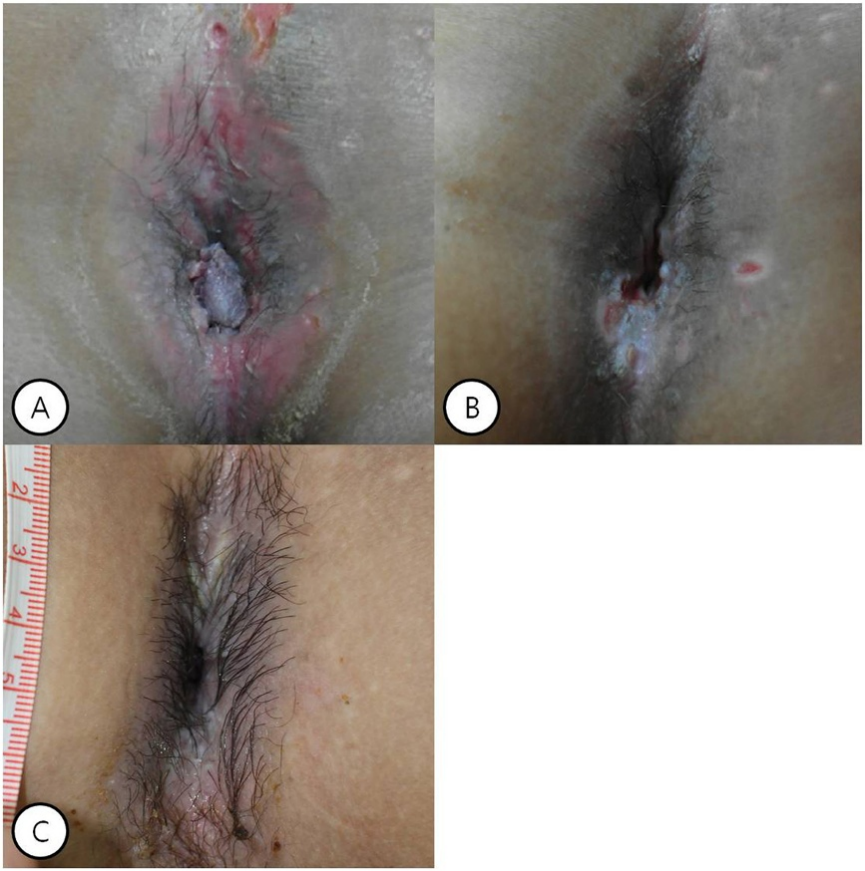
**Gross finding of giant condyloma acuminatum of anus. (A)** Six weeks after local therapy, the mass markedly decreased and was only present in the anal canal. **(B)** The mass was removed by local surgical excision under general anesthesia, and then bleomycin irrigation was done twice. **(C)** At seven months, there was no mass in the perianal and anal orifices.

**Figure 3:**
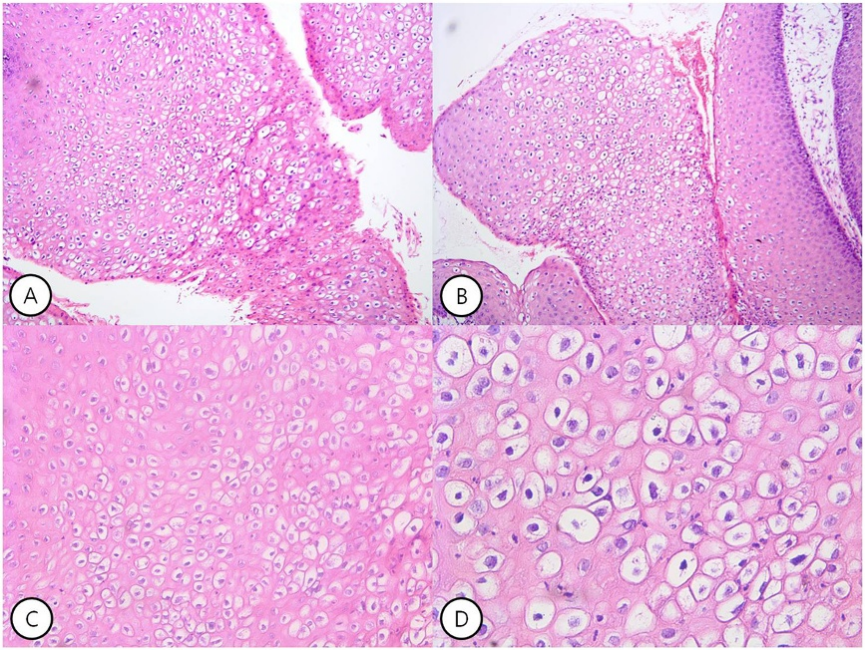
**Pathologic finding of giant condyloma acuminatum of anus. (A)**, **(B)** The epithelium shows considerable superficial hyperkeratosis with koilocytosis (hematoxylin and eosin staining magnification ×100). **(C)** There is distinct perinuclear clear vacuolization of the prickle cells (koilocytosis) (hematoxylin and eosin staining; magnification ×200). **(D)** Koilocytosis characteristic of HPV infection (hematoxylin and eosin; magnification ×400).

A 31-year-old Korean male presented to our hospital with anal discomfort for more than one month due to a protruding mass. He had a history of BCR-ABL-positive acute lymphoblastic leukemia (ALL) and had undergone an allogenic stem cell transplantation from a human leukocyte antigen (HLA) 1 locus-mismatched unrelated donor approximately 70 days prior. The conditioning regimen was busulfan, fludarabine, antithymocyte globulin (BU-FLU-ATG), and he had been tapered on cyclosporine for prophylaxis of graft versus host disease (GVHD). He was at an especially high risk of GVHD, therefore we used total 9mg/kg of ATG for strong immunosuppression. On post-transplant day 69, he complained of discomfort due to an anal mass, which gradually grew over the course of one month.Gross findings revealed a large perianal cauliflower-like mass over 7cm in size with invasion of the anal orifice (Figure 1-A). He was not married and did not have a history of anal intercourse, furthermore he had not any intercourse within one year due to induction and consolidative chemotherapy for ALL. He tested negative for HIV and venereal disease research laboratories test (VDRL). Based on the data, he was diagnosed with giant anal condyloma of anus occurring after an allogeneic bone marrow transplantation.He was initially treated with podophyllin and cryotherapy under the care of a dermatologist. Because of the unusual size of the mass, he was treated a total of six times per week (Figure 1B, C, D). After treatment, the mass markedly decreased and was only present in the anal canal (Figure 2A). The mass was removed by surgical excision using scissors and electrocautery under general anesthesia. Postoperative histopathologic examination confirmed a condyloma without malignant transformation or dysplasia (Figure 3). He tested positive for HPV 6 and negative for HPV 11, 16, and 18. Following removal of the condyloma mass, thereafter followed a bleomycin local injection into the small remnant lesion. We performed the irrigation twice with bleomycin mixed with 0.9% normal saline, at a final concentration of 1.0U/mL (Figure 2B). On post-transplant day 90, cyclosporine treatment was stopped. After seven months, there was no mass present in the perianal and anal orifices, and there were no problems in defecation. He continues to have normal anal function. (Figure 2C).

Successful treatment included a combination of topical podophyllin and cryotherapy and transanal surgical excision, followed by bleomycin irrigation.

## Discussion

GCA is a slow growing cauliflower-like tumor, however, it is locally invasive, destructive, and may exceed 10cm in diameter [4]. It is a rare sexually transmitted disease that affects the anogenital region, with an incidence of approximately 0.1% in the general population [5, 6]. HPV (6, 11, 16, and 18) has been found in biopsied tissue from condylomata acuminata. HPV 6 and 11 are the two most common subtypes and are usually nononcogenic or low risk. The presence of HPV 16 and 18 within a condyloma increases the oncogenic potential and is typically associated with carcinomas of the penis and anus [7, 8]. HPV can be transmitted via several pathways: sexual contact, autoinoculation, or contact with infected materials. The incubation period is usually two to three months, but can last for up to 20 months. The anal region can be used for sexual satisfaction, especially for homosexual men. The prevalence of homosexual intercourse has increased, as has the practice of receptive anal intercourse [9]. Other risk factors for the development of anal and/or perianal giant condyloma are immunosuppression, such as HIV infection, post-transplantation, chronic irritation due to perianal fistula and ulcerative colitis, and poor personal hygiene [4].

These lesions start as keratotic plaque and slowly expand into a cauliflower-like mass, forming a locally invasive, non-metastasizing growth that can nearly obliterate the anal canal. The symptoms at first presentation are primarily a mass or multiple masses. Macroscopically, the mass appears to be polypoid, cauliflower-like, and exophytic. It is characterized by slow growth, local infiltration, and contiguous tissue destruction, and has a high tendency to recur and produce fistulas or abscesses around the affected area. The most frequent areas of localization are the surface of vulva, the scrotum, the penis, the perineum, and the perianal region. Other symptoms including pain, fistula, abscess, persistent drainage, bleeding, pruritus, and difficulty walking and defecating may present alone or combined. Clinical examination has shown masses that are mobile toward the deep fascia and tissues. While both giant condyloma acuminatum and simple condylomas have a benign histology, the former is differentiated by a thicker stratum corneum, marked papillary proliferation, and a tendency to invade deep and displace underlying tissue with an intact basement membrane. As such, it is considered as an intermediate lesion between condyloma acuminatum and invasive squamous cell carcinoma [4–6].

In our case report, the giant condyloma was due to immunodeficiency (post-allogeneic transplantation). Immunosuppressed solid organ allograft recipients rarely develop anogenital lesions (2.3%), and giant condyloma after bone marrow transplantation is extremely rare (0.3 to 1.3%) [2, 3, 10]. Immunodeficiency is common after allogeneic stem cell transplantation. CD31 and CD41 T-cell counts remain depressed until two to three years post-transplant, whereas CD81 T-cell counts normalize by 18 months. Furthermore, human leukocyte antigen (HLA)-mismatching between the donor and the recipient and T-cell depletion can delay immune recovery. Ongoing immunosuppression in patients undergoing transplantation provides fertile ground for untamed proliferation of the HPV virus, which leads to a higher chance of dissemination, local invasion, and oncogenesis [2, 3, 11, 12]. To date, no definitive therapeutic strategies have been established [4, 5].

The majority of authors agree that surgery is the treatment of choice and is efficient in the early stages of the disease. Surgical excision is the first line of treatment for giant condyloma because of a relatively high success rate (63 to 91%) and low risk of recurrence. Excision of the surrounding area must be comprehensive [4, 5, 12]. However, other practitioners support conservative surgery as the best choice, mainly in terms of the patient’s quality of life [13]. Simple excision alone has been reported as a sufficient therapeutic strategy in several GCA cases without evidence of disease recurrence [14]. Podofilox solution and gel are other treatment options for condyloma acuminata. With the 0.5% solution, recurrences were found in one third of patients during the first month after treatment. A high risk of recurrence has been reported, likely because of the presence of the virus in adjacent, normal-appearing tissues [15]. Cryotherapy alone or in combination with topical 5-fluorouracil has been reported with good results in relatively small Buschke-Löwenstein tumors [16]. The use of radiotherapy is controversial, as verrucous carcinoma may transform into poorly differentiated carcinoma with subsequent metastases. CO_2_ laser therapy has been successfully applied [5, 17].

The antimetabolite 5-fluorouracil, bleomycin combined with cisplatin or methotrexate, and α-interferon in topical or systemic administration are possible management options to reduce the size of giant condylomas [5, 18, 19]. However, there are no guidelines or standard doses and treatments are expensive and require multiple administrations.

## Conclusions

We report an extremely rare case of GCA after an allergenic stem cell transplantation. We presume that the risk factor of rapid progression in our case report was strong immunosuppression due to post-allogeneic transplantation using a relative high dose of T lymphocyte depletion agent (ATG) and cyclosporine. Especially, another presumed risk factor may have been prolonged chronic irritation due to sitz baths with betadine solution over three times per day, leading the tumor to become giant in size. Although most case reports suggest broad excision as the initial standard treatment of giant condyloma, we successfully preserved anal function using a step-by-step combined modality with topical podophyllin and local cryotherapy and transanal surgical excision, followed by bleomycin irrigation.

## Consent

Written informed consent was obtained from the patient for publication of this case report and any accompanying images. A copy of the written consent is available for review by the Editor-in-Chief of this journal.

## Abbreviations

HPV: human papillomavirus
GCA: giant condyloma acuminatum
HIV: human immunodeficiency virus
ALL: acute lymphoblastic leukemia
BU-FLU-ATG: busulfan, fludarabine, antithymocyte globulin
GVHD: graft versus host disease
VDRL: venereal disease research laboratories test
HLA: leukocyte antigen.
